# Mortality Level and Predictors in a Rural Ethiopian Population: Community Based Longitudinal Study

**DOI:** 10.1371/journal.pone.0093099

**Published:** 2014-03-27

**Authors:** Berhe Weldearegawi, Mark Spigt, Yemane Berhane, GeertJan Dinant

**Affiliations:** 1 Department of Public Health, College of Health Sciences, Mekelle University, Mekelle, Ethiopia; 2 CAPHRI, School for Public Health and Primary Care, Maastricht University, Maastricht, Netherlands; 3 Addis Continental Institute of Public Health, Addis Ababa, Ethiopia; Institute of Neuroepidemiology and Tropical Neurology, France

## Abstract

**Background:**

Over the last fifty years the world has seen enormous decline in mortality rates. However, in low-income countries, where vital registration systems are absent, mortality statistics are not easily available. The recent economic growth of Ethiopia and the parallel large scale healthcare investments make investigating mortality figures worthwhile.

**Methods:**

Longitudinal health and demographic surveillance data collected from September 11, 2009 to September 10, 2012 were analysed. We computed incidence of mortality, overall and stratified by background variables. Poisson regression was used to test for a linear trend in the standardized mortality rates. Cox-regression analysis was used to identify predictors of mortality. Households located at <2300 meter and ≥2300 meter altitude were defined to be midland and highland, respectively.

**Results:**

An open cohort, with a baseline population of 66,438 individuals, was followed for three years to generate 194,083 person-years of observation. The crude mortality rate was 4.04 (95% CI: 3.77, 4.34) per 1,000 person-years. During the follow-up period, incidence of mortality significantly declined among under five (P<0.001) and 5–14 years old (P<0.001), whereas it increased among 65 years and above (P<0.001). Adjusted for other covariates, mortality was higher in males (hazard ratio (HR) = 1.42, 95% CI: 1.22, 1.66), rural population (HR = 1.74, 95% CI: 1.32, 2.31), highland (HR = 1.20, 95% CI: 1.03, 1.40) and among those widowed (HR = 2.25, 95% CI: 1.81, 2.80) and divorced (HR = 1.80, 95% CI: 1.30, 2.48).

**Conclusions:**

Overall mortality rate was low. The level and patterns of mortality indicate changes in the epidemiology of major causes of death. Certain population groups had significantly higher mortality rates and further research is warranted to identify causes of higher mortality in those groups.

## Introduction

Mortality is one of the most important indicators of the health status of a population [1], [2]. Mortality statistics stratified by age, sex and the cause of death are of great value for the formulation, implementation and evaluation of public health programs [3].

The presence of well-established civil registration systems enabled developed countries to monitor changes in mortality, determine causes of death and devise appropriate interventions [4]. Yet, vital registration systems are lacking in low-income countries, especially in Sub-Saharan Africa [5]. This hampers the evaluation of the health status of populations and the impact of interventions [5]. Since it is unreasonable to expect an immediate implementation of nationwide population-based registration systems in low-income countries, considering other interim options is important. One way is to select a circumscribed population from which reasonably detailed, complete, and high quality community-based data can be gathered longitudinally, the so called Health and Demographic Surveillance System (HDSS) [6], [7]. Typical HDSS populations include at least 60,000 individuals, which is usually sufficient to provide adequate sample sizes to monitor trends in mortality [5].

Ethiopia, the second most populous country in Africa, has implemented an ambitious economic development plan and a twenty-year Health Sector Development Plan (HSDP) to improve access and utilization of health care services [8]–[10]. Moreover, Ethiopia is undergoing rapid economic growth, urbanization, and change in life-style and nutrition transition [11]–[13]. Monitoring the effects of these countrywide changes would not only help to understand the Ethiopian situation. It will be very informative to countries who plan to implement similar initiatives, and it will show to the world which changes in mortality we can expect if developing countries are changing. The Kilite Awlaelo HDSS (KA-HDSS) was established in September 2009 to generate population based longitudinal health and demographic information. This gave us the unique opportunity to investigate the mortality levels, patterns and the predictors of mortality in a predominantly rural low-income population.

## Methods

**Figure 1:**
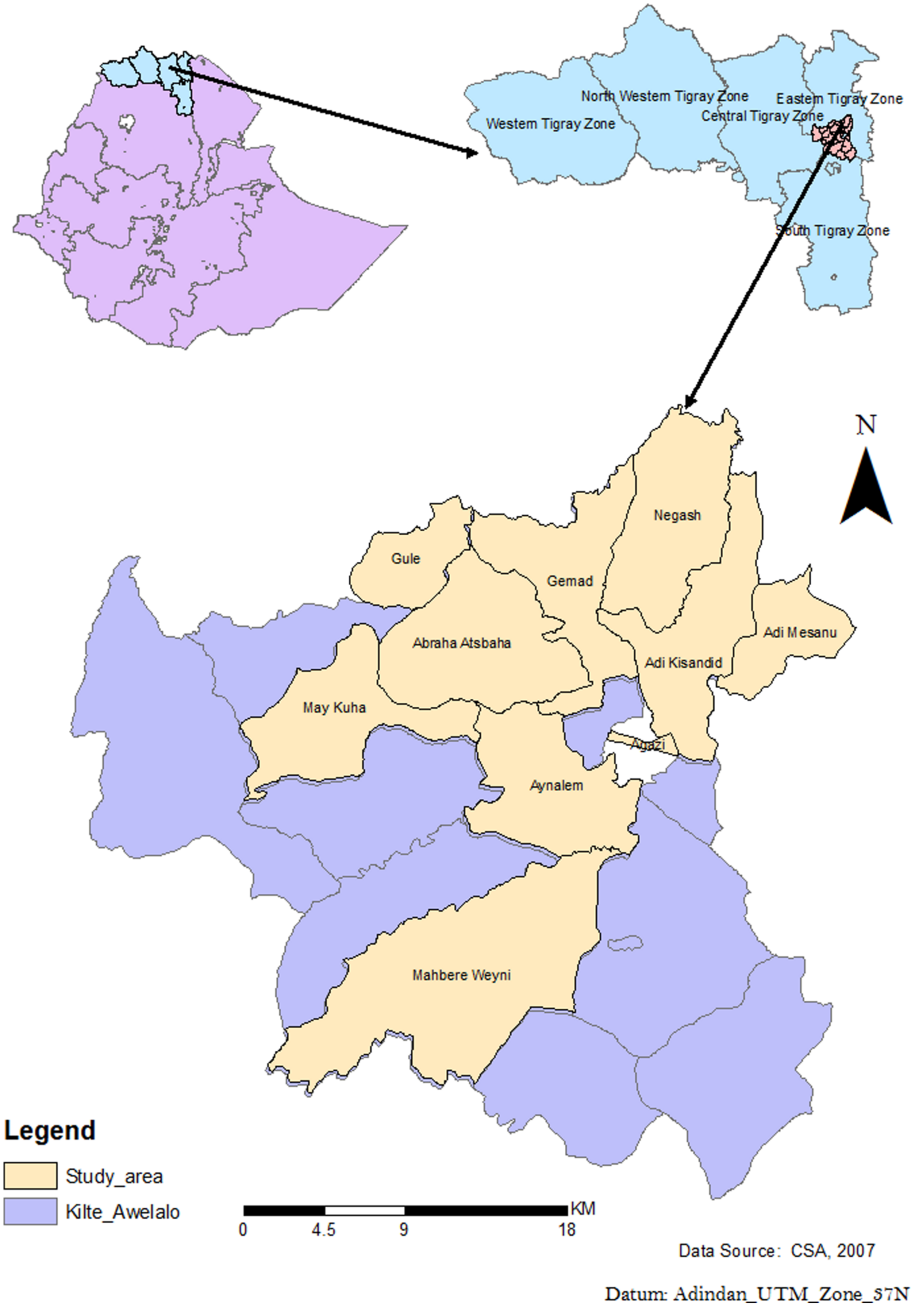
Map of Ethiopia, Tigray and the kilite Awlaelo HDSS.

This study used data generated by the KA-HDSS, which is a longitudinal population-based surveillance system. The KA-HDSS, member of the INDEPTH Network [14], is located about 802 km North of Addis Ababa, the capital of Ethiopia. Nine rural and one urban Kebele (smallest administrative unit in Ethiopia with average population of 5,000) were selected using the probability proportional to size technique (Figure 1). Agro-climatic condition, rural-urban composition, geographic location (highland and midland) and disease burden considerations were made during selection of study villages.

The cohort was established with baseline data from 66,438 individuals living in 14,453 households. All households in the selected Kebeles and all individuals in these households were included in the follow-up that was done twice in a year through house-to-house visit. During each visit, vital event information on pregnancy status, birth, cause of death with verbal autopsy [15], marital status change, and migrations were collected. Full time data collectors, who at least completed high school, were recruited from the surveillance kebeles. They were trained for five days on data collection tools, interviewing techniques and ethical conduct of research using standard field manual. Besides, they were provided with refresher training biannually. The data collection process was supervised by field supervisors, a field coordinator and the research team.

To link event histories, a permanent unique identification number (ID) was given for each individual and household that ever entered the cohort. To avoid incorrect attribution of data, household and individual ID were neither given to another individual or household nor changed over time. The surveillance employed standard data collection tools and procedures adopted from the INDEPTH Network [14]. Geographic location data were also collected at household level. Households located at <2300 meter and ≥2300 meter altitude were defined to be a midland and highland, respectively [16]. All study households had access to primary health care facilities (with in 5 km distance), that provide free maternal and child health services. At kebele level, there are two Health Extension Workers (HEWs) who are responsible for health promotion, prevention and treatment of common illnesses.

The KA-HDSS uses the Household Registration System (HRS version 2.1) FoxPro database. Data analysis was done using STATA 11. Incidence of mortality was calculated by dividing number of deaths in a given group or time period by the total sum of person-time in the specific group or time period. Person-time of observation was determined as the difference between a subject's end date and start date of follow-up. The total person-time was split by year and age-category to calculate mortality rates by age and by year. Cox proportional hazards regression model were used to estimate hazard ratios and corresponding 95% confidence intervals. Poisson regression was used to test for a linear trend in the standardized mortality rates. This paper is based on three years surveillance data, from September 11, 2009 to September 10, 2012.

### Ethical Statement

The KA-HDSS received ethical clearance from the Ethiopian Science and Technology Agency with identification number IERC 0030. Ethical approval, with reference number ERC 0377/2014, was also obtained from the Health Research Ethics Review Committee (HRERC) of Mekelle University. To capture occurrence of vital events to any family member, head of a family or an eligible adult among the family was interviewed. Therefore, informed verbal consent was obtained from head of the family or eligible adult among the family, rather than each subject. This consent procedure was stated in the proposal which was approved by the ethical review committee. To keep confidentiality, data containing personal identifiers of subjects were not shared to third party.

## Results

The population under surveillance was relatively young; with 39.7% of it aged less than 15 years old, and only 5.3% aged 65 years and above. The population composition showed a male: female sex ratio of 0.93, with a slight overrepresentation of females. About 88.2% of the total population lived in rural districts. The age group 15–19 years and 55–59 years contributed the highest and lowest person-years of observation, contributing 15.5% and 2.2% of the total person-time observed, respectively (table 1).

**Table 1:** Person-years of follow-up and number of deaths to the KA-HDSS cohort, September 11, 2009–September 10, 2012.

Characteristics	Person-years of follow-up			Number of deaths		
	Female	Male	Total	Female	Male	Total
**Age in years**						
<5	6871	7170	14044	64	82	146
5–9	13631	14234	27872	12	18	30
10–14	14519	14867	29394	7	16	23
15–19	14783	15381	30171	9	22	31
20–24	10236	10043	20284	11	11	22
25–29	6518	5170	11692	11	9	20
30–34	6737	3935	10675	15	12	27
35–39	5248	3447	8698	9	10	19
40–44	4669	3851	8522	5	14	19
45–49	2411	2632	5044	7	8	15
50–54	4249	2846	7097	22	12	34
55–59	2312	1916	4229	12	3	15
60–64	2902	2658	5561	23	25	48
65+	4997	5851	10851	154	182	336
**Geographic location**						
Midland	73595	69545	143178	248	290	538
Highland	26488	24454	50956	112	135	247
**Residence**						
Urban	13941	10614	24562	31	25	56
Rural	86142	83385	169571	330	399	729
**Marital status**						
Married	26833	24890	51737	70	197	267
Single	33876	37766	71660	51	70	121
Divorced	4963	982	5948	29	18	47
Widowed	6018	842	6863	131	36	167
Under age*	28393	29519	57927	80	103	183
**Sex**						
Female			100083			361
Male			93999			424

*under 10 years old.

A total of 194,083 person-years were generated during the three years follow-up period and 785 deaths occurred over the same period resulting in a crude mortality rate of 4.04 per 1,000 person-years of observation, with a 95% confidence interval (CI) (3.77, 4.34). Table 2 summarizes mortality rates by background variables. It shows that mortality was on average higher among males (4.55 versus 3.64 per 1,000 person-years). Mortality was highest among the oldest age group (65+ years) and under-five children, while it was lowest among of 5–14 years old. Rural residents had a mortality rate of twice higher than their urban counterparts; 4.34 versus 2.30 respectively. With regard to marital status, death rates were noticeably higher in those divorced and those widowed; 8.01 per 1,000 person-years and 24.68 per 1,000 person-years, respectively. Sex differences were also noticed; divorced males had a three times higher and widowed males a twice higher mortality rate compared to females of the same marital status.

**Table 2:** Mortality rates by background and geographic variables, the KA-HDSS cohort, September 11, 2009-September 10, 2012.

Characteristics	Female	Male	Overall
	Rate (95%CI)	Rate (95%CI)	Rate (95%CI)
**Sex**			
Female			3.64 (3.29, 4.04)
Male			4.55 (4.14, 5.01)
**Age in years**			
Under 5	5.51 (4.28, 7.10)	6.08 (4.79, 7.71)	5.80 (4.88, 6.90)
5–14	0.74 (0.47, 1.16)	1.09 (0.75, 1.56)	0.92 (0.69, 1.22)
15–49	1.36 (1.07, 1.73)	2.07 (1.68, 2.55)	1.69 (1.45, 1.98)
50–64	5.94 (4.57, 7.72)	5.17 (3.77, 7.11)	5.61 (4.58, 6.86)
65+	31.69 (27.08, 37.10)	32.27 (27.94, 37.27)	32.01 (28.78, 35.59)
**Marital status**			
Married	2.63 (2.08, 3.33)	7.98 (6.94, 9.18)	5.21 (4.62, 5.87)
Single*	1.52 (1.15, 2.00)	1.87 (1.48, 2.36)	1.70 (1.43, 2.04)
Divorced	5.91 (4.10, 8.50)	18.82 (11.85, 29.86)	8.01 (6.02, 10.66)
Widowed	22.02 (18.56, 26.14)	44.03 (31.76, 61.04)	24.68 (21.21, 28.73)
Underage	2.85 (2.29, 3.54)	3.52 (2.91, 4.28)	3.19 (2.76, 3.69)
**Residence**			
Urban	2.24 (1.58, 3.19)	2.38 (1.61, 3.52)	2.30 (1.77, 2.99)
Rural	3.87 (3.47, 4.31)	4.83 (4.38, 5.33)	4.34 (4.04, 4.67)
**Geographic location**			
Midland	3.39 (2.99, 3.84)	4.21 (3.75, 4.72)	3.79 (3.48, 4.12)
Highland	4.29 (3.57, 5.17)	5.52 (4.65, 6.54)	4.88 (4.31, 5.53)

All rates are per 1000 person-years.

* adults who are never married before.

All predictors variables tested in the univariate Cox-regression were significant, and also in the multivariate model; adjusted for age, sex, residence, geographic location and marital status. As shown in table 3, except the old age group (65+), all other age groups had lower mortality compared to under five. The hazard ratio (HR) for mortality in males compared with females was 1.42 (95% CI: 1.22, 1.66). Being widowed and divorced were significantly associated with higher hazard of death (HR = 2.25, 95%CI: 1.81, 2.80) and (HR = 1.80, 95%CI: 1.30, 2.48) respectively. Rural residence and highland geographic location also predicted mortality with statistical significance.

**Table 3:** Multivariate Cox-regression model for predictors of mortality, using a, the KA-HDSS cohort, September 11, 2009–September 10, 2012.

Characteristics	Crude HR (95%CI)	Adjusted HR (95%CI)
**Age in years**		
Under 5	1.00	1.00
5–14	0.09 (0.06, 0.12)	0.09 (0.06, 0.12)**
15–49	0.15 (0.12, 0.19)	0.13 (0.07, 0.23)**
50–64	0.55 (0.42, 0.71)	0.37 (0.20, 0.70)**
65+	2.95 (2.43, 3.58)	1.74 (0.95, 3.20)
**Sex**		
Female	1.00	1.00
Male	1.25 (1.09, 1.44)	1.42 (1.22, 1.66)**
**Residence**		
Urban	1.00	1.00
Rural	1.88 (1.44, 2.47)	1.74 (1.32, 2.31)**
**Marital status**		
Married	1.00	1.00
Single	0.33 (0.26, 0.41)	0.92 (0.70, 1.20)
Divorced	1.54 (1.13, 2.10)	1.80 (1.30, 2.48)**
Widowed	4.73 (3.90, 5.75)	2.25 (1.81, 2.80)**
Underage	0.61 (0.51, 0.74)	0.81 (0.45, 1.46)
**Geographic location**		
Midland	1.00	1.00
Highland	1.29 (1.11, 1.50)	1.20 (1.03, 1.40)*

HR: Hazard ratio.

* Significant at P<0.05.

** Significant at P<0.01.

The age-adjusted death rate was comparable over the three years follow-up period, with the highest 4.23 per 1,000 person-years observed during the second follow-up year (table 4). Mortality rate observed by age category showed a declining pattern among younger ages and increasing in people in the old age group. During the follow-up period, incidence of mortality significantly declined among under five (P<0.001) and 5–14 years (P<0.001), whereas it increased among 65 years and above (P<0.001). There was also a significant mortality decline in the midlands (P = 0.001), while the increase in highlands was not significant (P = 0.33).

**Table 4:** Trend in standardized mortality rate, the KA-HDSS cohort, September 11, 2009–September 10, 2012.

Characteristics	Year I	Year II	Year III	P-value*
	Rate (95%CI)	Rate (95%CI)	Rate (95%CI)	
**Age in years**				
<5	0.77 (0.55, 0.99)	0.65(0.49, 0.82)	0.36 (0.25, 0.47)	<001
5–14	0.38 (0.22, 0.53)	0.24 (0.12, 0.36)	0.21 (0.10, 0.33)	0.007
15–49	0.77 (0.55, 0.99)	0.90 (0.67, 1.14)	0.79 (0.57, 1.02)	0.88
50–64	0.39 (0.24, 0.55)	0.59 (0.41, 0.78)	0.53 (0.35, 0.70)	0.83
65+	1.66 (1.34, 1.98)	1.84 (1.51, 2.17)	1.91 (1.57, 2.25)	<001
Overall	3.80 (3.48, 4.47)	4.23 (3.74, 4.72)	3.80 (3.33, 4.27)	0.08
**Location**				
Midland	4.03 (3.49, 4.67)	3.89 (3.37, 4.48)	3.46 (2.97, 4.02)	0.001
Highland	3.72 (2.88, 4.80)	5.69 (4.67, 6.94)	5.14 (4.17, 6.34)	0.33

* P-value represents test for linear trend in stand.ardized rates.

Year I: September 11, 2009–september 10, 2010.

Year II: September 11, 2010–september 10, 2011.

Year III: September 11, 2011–september 10, 2012.

## Discussion

The mortality rate reported in this large prospective study was low. The identified predictors of mortality; age, sex, residence and marital status were as expected, except for the higher risk of mortality in highland areas. During the follow-up period, mortality rate declined in younger ages while it increased in elderly people. The trend in age specific mortality over time supports the idea of epidemiologic transition in low-income countries. In low-income countries, which are undergoing economic development and change in lifestyle, such changes in mortality patterns could be expected.

Though comparison may be affected by methodological variation, our observed mortality rate was much lower than the extrapolated estimates for Ethiopia (9 per 1,000) and Sub-Saharan countries like: Kenya (10 per 1,000), Eritrea (8 per 1,000), Sudan (9 per 1,000) and Tanzania (10 per 1,000) [17]. Findings from HDSS sites employing similar methodology also showed higher mortality rates than our findings, while others reported comparable figures [18]–[23]. The HDSS sites; Navrongo (Ghana), Mbita and Kwale (Kenya), Kersa (Ethiopia) and Butajira (Ethiopia) reported more than twice higher mortality rate than findings of the current study (ranging from 9–13 per 1,000). Other HDSS sites like; Kilifi (Kenya) and Gigel Gibe (Ethiopia) had a similar mortality rate (ranging from 5.8–7.7 per 1,000).

Though, the mortality level reported in the current study is not claimed to represent the exact estimates for Ethiopia, the observed mortality level would be expected; considering the recent economic development, improvements in health care and other social services [8]–[10], [24], [25]. Primary health service coverage has nowadays reached 92% [26]. According to the world bank, the economy has experienced strong and broad based growth over the past decade, averaging 9.9% per year in 2004/05 - 2011/12 compared to the East African average of 5.4% [17]. Moreover, the districts included in the surveillance and the region as whole, has served as a pilot area for new health policies like the health extension program, women development army and health insurance [27].

The decline in under five mortality during the follow-up period, was significant. Though such a sharp decline in a short period is uncommon, decline in under five mortality is reported at both national and regional levels [9], [28]. This sharp decline in under five mortality could possibly be explained by the introduction of Pneumococcal Conjugate Vaccine (PCV-10) in September 2011, and improvements in antenatal care and skilled birth attendance, which may have reduced neonatal and child mortality [27]. On the other hand, the emerging burden of non-communicable diseases and relative neglect of adult and old age health in developing countries could explain the increasing pattern of mortality among old age groups [29], [30].

Several reports support the mortality disadvantage of males reported in the current study [31]–[33]. In many societies, male engage in more dangerous, stressful or difficult occupations than women [33], [34]. A previous study on the same cohort reported that external causes (accidents, injuries), which are the commonest causes of death in the study area, were twice more common in males [35]. As has consistently been documented, rural residents had a mortality disadvantage compared to their urban counterparts [28]–[31]. This is likely to be associated with comparative disadvantages of rural population in economic, health care and other social services [36].

Adjusted for other covariates, widowed and divorced persons had a higher mortality compared to those who were married. The lower longevity of widowed and divorced people has been repeatedly documented [30], [33], [37]–[40]. Males that belong to either of these two categories of marital status were more affected than females. A large cohort study in Japan also supports this finding [40]. There are three models that explain the longevity of married people; the resource, stress and selection models; all demonstrate the selective nature and protective effects of marriage [32], [39]. Though it might be expected that the higher mortality in those widowed and divorced groups could be related to epidemiology of HIV/AIDS in those groups, findings showed higher mortality levels from all causes of death, major cardiovascular causes and external causes of death among those groups [39], [40]. The disadvantage in risk of death in those widowed and divorced male is explained in terms of the differences in benefits from marriage. In general, male benefit more from marriage in terms of social support and social control of health behaviors than female [39].

The higher hazard of mortality in highlands was inconsistent to reports of other studies [20], [41], [42]. Though further investigations are warranted, the inconsistency can be explained in terms of change in the epidemiology of major causes of death [30], [35]. A previous study in the same population reported that mortality from infectious and parasitic causes account for 36% of total deaths while chronic non-communicable diseases and external causes of death accounted for 29% and 16%, respectively. The burden of malaria, which usually affects people living the lowland areas, is also low [35].

Our study has several strengths. The mortality figure in our report is unlikely to be due to missing event registration. The data collection process is undertaken under strict supervision and follow-up. Moreover, as follow-up starts when a mother is pregnant, the probability of missing deaths immediate after birth is minimal, while missing highly mourned adult death is unlikely. Furthermore, a study on robustness of the HDSS surveillance data showed that even a random error of about 20% introduced has no significant effect on the parameter estimates and regression analyses [43]. In addition, the KA-HDSS uses standardized data collection tools and procedures, which ensure the quality of data. The study has also limitations. Despite the efforts to ensure representativeness during selection of the study villages, we can not claim that the current findings are nationally representative; because many factors like: access and utilization of social services and lifestyle that affect health status varies in various parts of the country. Background variables such as socio-economic status and climate change, which might help further explanation, were not collected. The short follow-up period may also restrict the usage of the current estimate in predicting mortality patterns.

In conclusion, this study reported a lower overall mortality rate in a predominantly rural population. Yet, differentials in mortality are observed by socio-demographic and geographic location. Moreover, the excess mortality in highland, declining mortality in younger ages, and the increasing mortality in people of old age highlights changes in the epidemiology of major causes of death. Even though the overall mortality is low, the observed disparities should be considered during planning for health interventions and social services. Certain population groups had significantly higher mortality rates and further research is warranted to identify causes of higher mortality in those groups.
